# Importance of twitching and surface-associated motility in the virulence of *Acinetobacter baumannii*

**DOI:** 10.1080/21505594.2021.1950268

**Published:** 2021-09-13

**Authors:** Jordi Corral, María Pérez-Varela, Miquel Sánchez-Osuna, Pilar Cortés, Jordi Barbé, Jesús Aranda

**Affiliations:** Departament De Genètica I Microbiologia, Facultat De Biociènces, Universitat Autònoma De Barcelona, Barcelona, Spain

**Keywords:** *A. baumannii*, twitching, surface-associated motility, virulence

## Abstract

*Acinetobacter baumannii* is a pathogen of increasing clinical importance worldwide, especially given its ability to readily acquire resistance determinants. Motile strains of this bacterium can move by either or both of two types of motility: (i) twitching, driven by type IV pili, and (ii) surface-associated motility, an appendage-independent form of movement. *A. baumannii* strain MAR002 possesses both twitching and surface-associated motility. In this study, we isolated spontaneous rifampin-resistant mutants of strain MAR002 in which point mutations in the *rpoB* gene were identified that resulted in an altered motility pattern. Transcriptomic analysis of mutants lacking twitching, surface-associated motility, or both led to the identification of deregulated genes within each motility phenotype, based on their level of expression and their biological function. Investigations of the corresponding knockout mutants revealed several genes involved in the motility of *A. baumannii* strain MAR002, including two involved in twitching (encoding a minor pilin subunit and an RND [resistance nodulation division] component), one in surface-associated motility (encoding an amino acid permease), and eight in both (encoding RND and ABC components, the energy transducer TonB, the porin OprD, the T6SS component TagF, an IclR transcriptional regulator, a PQQ-dependent sugar dehydrogenase, and a putative pectate lyase). Virulence assays showed the reduced pathogenicity of mutants with impairments in both types of motility or in surface-associated motility alone. By contrast, the virulence of twitching-affected mutants was not affected. These results shed light on the key role of surface-associated motility and the limited role of twitching in the pathogenicity of *A. baumannii*.

## Introduction

*Acinetobacter baumannii* is a gram-negative bacterium that causes nosocomial infections in hospitals worldwide, mainly ventilator-associated and bloodstream infections in critically ill patients [1]. The pathogenic success of this bacterium can be attributed to its capacity to survive in healthcare environments, its potential to acquire and develop antimicrobial resistance, and to its virulence [2]. In response to the increasing clinical importance of multidrug-resistant bacteria, the World Health Organization created a priority list of antibiotic-resistant bacterial species, including *A. baumannii*, to encourage research into effective drugs and the development of novel therapeutic strategies[3].

While the name *Acinetobacter* comes from the Greek word “*akineto*,” translated as “non-motile,” many strains of *A. baumannii* are in fact motile, through two different mechanisms: twitching motility and surface-associated motility. Twitching motility is a coordinated multicellular movement driven by the extension, attachment, and retraction of type IV pili [4,5]. It is associated with surface attachment and biofilm formation in a process that includes the pilA, pilD, and pilT genes [4,6] and the GacS/GacA two-component regulatory system [7]. Surface-associated motility is an appendage-independent form of movement characteristic of some A. baumannii clinical isolates and probably driven by the extrusion of extrapolymeric substances [8,9]. This type of motility is associated with the synthesis of 1,3-diaminopropane [8], lipooligosaccharide production [9], proteins related to metabolism, the outer membrane, and natural competence [10], and proteins from at least five of the six known superfamilies of efflux pumps [11]. Regulators of surface-associated motility include quorum sensing [12], cyclic diguanylate [13], blue-light sensing [14], CheW and CheA chemotactic analogs [15,16], and the EnvZ/OmpR two-component system [17]. The capacity to move by twitching or surface-associated motility depends on the A. baumannii strain. Thus, strain ATCC 17978 exhibits only surface-associated motility, strain AYE only twitching, strain MAR002 is capable of both, and other strains, such as ATCC 19606, are non-motile [18,19].

]In many different bacterial species, virulence is closely related to motility, as is the case for *A. baumannii* [2,20]. This association suggests that bacterial motility can be exploited as a specific preventive or therapeutic antibacterial target, with the aim of either cure or disease prevention [21]. Although the role of twitching motility in virulence is so far unknown [2], a key role for surface-associated motility in the virulence of *A. baumannii* has been demonstrated in several reports [8,10,11,16,22,23].

Point mutations in the bacterial *rpoB* gene, encoding the β-subunit of the RNA polymerase, confer rifampin resistance and cause substantial changes in the transcriptional profile of bacterial cells, by affecting the enzyme’s affinity for several promoters [24–26]. Rifampin inhibits DNA-dependent RNA synthesis through its interaction with the RpoB subunit. Mutations in the *rpoB* gene that produce conformational changes in the region of rifampin interaction reduce the affinity of RpoB for this antimicrobial and thus confer resistance. Moreover, the new conformation of RpoB modifies the polymerase’s affinity for certain promoters, causing changes in gene expression[27]. In other bacterial species, such as *Escherichia coli* or *Pseudomonas aeruginosa*, mutations in the *rpoB* gene induce pleiotropic effects that impact processes such as sporulation[28], stringent response [25,29], motility, and virulence [24,30].

In a previous study conducted in *A. baumannii* strain ATCC 17978, which possesses only surface-associated motility, we showed that point mutations in *rpoB* impair both motility and virulence[23]. A transcriptional analysis of these spontaneous rifampin-resistant mutants led to the identification of six genes encoding proteins homologous to the transporters and metabolic enzymes involved in the surface-associated motility of *A. baumannii*[23].

In the present work, spontaneous rifampin-resistant mutants of *A. baumannii* strain MAR002, a biofilm-hyperproducing strain isolated from a wound sample collected from a patient in Spain[31] and possessing both twitching and surface-associated motility, were isolated. Three different motility-related phenotypes were identified among the mutants: (i) twitching deficient, (ii) surface-associated motility deficient, and (iii) lacking both movement phenotypes. A transcriptional analysis of these mutants allowed us to identify genes involved in either or both forms of motility. Experiments with the respective knockout mutants revealed the involvement of several of these genes in virulence as well.

## Materials and methods

### Bacterial strains, plasmids and growth conditions

The bacterial strains and plasmids used in this study are listed in Table 1. *A. baumannii* strain MAR002 and *E. coli* strain DH5α were grown at 37°C in Luria-Bertani (LB) medium[32] with shaking at 180 rpm. The pCR-BluntII-TOPO (Invitrogen, catalog number 451,245) and pBAV1Gm-T5-gfp plasmids were used for mutant construction and complementation, respectively (Table 1). When needed, rifampin (50 mg/L), kanamycin (50 mg/L), or gentamicin (20 mg/L for *E. coli* and 40 mg/L for *A. baumannii*) was added to the growth medium. *A. baumannii* growth was monitored using bacterial cultures in LB broth inoculated with an overnight culture at a dilution of 1:100 and then incubated at 37°C with shaking at 180 rpm. The optical density at 600 nm (OD_600_) of the cultures was measured hourly. To obtain rifampin-resistant (Rif^r^) mutants, saturated cultures of wild-type (WT) *A. baumannii* strain MAR002 were plated on rifampin-containing LB plates and incubated at 37°C for 24 h.

**Table 1.** Bacterial strains and plasmids used in this work

Strains or plasmids	Relevant characteristics	Source or reference
***E. coli*** strain		
DH5α	*E. coli supE4* Δ*lacU169* (*ɸ80* Δ*lacZ* Δ*M15*) *hsdR17, recA1, endA1, gyrA96, thi-1, relA1*	Clontech
***A. baumannii* strains**		
MAR002	Wild-type, biofilm-hyperproducing strain isolated from a wound sample	^[31]^
T^−^S^−^	Spontaneous rifampin-resistant MAR002 derivative strain with an impairment in twitching and surface-associated motility, Rif^r^	This work
T^+^S^−^	Spontaneous rifampin-resistant MAR002 derivative strain with an impairment in twitching motility, Rif^r^	This work
T^−^S^+^	Spontaneous rifampin-resistant MAR002 derivative strain with an impairment in surface-associated motility, Rif^r^	This work
*LH92_RS00045*	MAR002 derivative strain with *LH92*_*RS00045*::pCR-BluntII-TOPO disruption, Km^r^, Zeo^r^	This work
*LH92_RS01675*	MAR002 derivative strain with *LH92*_*RS01675*::pCR-BluntII-TOPO disruption, Km^r^, Zeo^r^	This work
*LH92_RS02585*	MAR002 derivative strain with *LH92*_*RS02585*::pCR-BluntII-TOPO disruption, Km^r^, Zeo^r^	This work
*LH92_RS04440*	MAR002 derivative strain with *LH92*_*RS04440*::pCR-BluntII-TOPO disruption, Km^r^, Zeo^r^	This work
*LH92_RS04440* complemented	*LH92_RS04440* strain carrying the pBAV1K-T5-gfp plasmid with the corresponding gene cloned, Km^r^, Zeo^r^, Gm^r^	This work
*LH92_RS05285*	MAR002 derivative strain with *LH92*_*RS05285*::pCR-BluntII-TOPO disruption, Km^r^, Zeo^r^	This work
*LH92_RS05285* complemented	*LH92_RS05285* strain carrying the pBAV1K-T5-gfp plasmid with the corresponding gene cloned, Km^r^, Zeo^r^, Gm^r^	This work
*LH92_RS05405*	MAR002 derivative strain with *LH92*_*RS05405*::pCR-BluntII-TOPO disruption, Km^r^, Zeo^r^	This work
*LH92_RS05405* complemented	*LH92_RS05405* strain carrying the pBAV1K-T5-gfp plasmid with the corresponding gene cloned, Km^r^, Zeo^r^, Gm^r^	This work
*LH92_RS06300*	MAR002 derivative strain with *LH92*_*RS06300*::pCR-BluntII-TOPO disruption, Km^r^, Zeo^r^	This work
*LH92_RS06300* complemented	*LH92_RS06300* strain carrying the pBAV1K-T5-gfp plasmid with the corresponding gene cloned, Km^r^, Zeo^r^, Gm^r^	This work
*LH92_RS06715*	MAR002 derivative strain with *LH92*_*RS06715*::pCR-BluntII-TOPO disruption, Km^r^, Zeo^r^	This work
*LH92_RS06715* complemented	*LH92_RS06715* strain carrying the pBAV1K-T5-gfp plasmid with the corresponding gene cloned, Km^r^, Zeo^r^, Gm^r^	This work
*LH92_RS07135*	MAR002 derivative strain with *LH92*_*RS07135*::pCR-BluntII-TOPO disruption, Km^r^, Zeo^r^	This work
*LH92_RS08005*	MAR002 derivative strain with *LH92*_*RS08005*::pCR-BluntII-TOPO disruption, Km^r^, Zeo^r^	This work
*LH92_RS12010*	MAR002 derivative strain with *LH92*_*RS12010*::pCR-BluntII-TOPO disruption, Km^r^, Zeo^r^	This work
*LH92_RS13540*	MAR002 derivative strain with *LH92*_*RS13540*::pCR-BluntII-TOPO disruption, Km^r^, Zeo^r^	This work
*LH92_RS13540* complemented	*LH92_RS13540* strain carrying the pBAV1K-T5-gfp plasmid with the corresponding gene cloned, Km^r^, Zeo^r^, Gm^r^	This work
*LH92_RS13935*	MAR002 derivative strain with *LH92*_*RS13935*:: pCR-BluntII-TOPO disruption, Km^r^, Zeo^r^	This work
*LH92_RS13935* complemented	*LH92_RS13935* strain carrying the pBAV1K-T5-gfp plasmid with the corresponding gene cloned, Km^r^, Zeo^r^, Gm^r^	This work
*LH92_RS15870*	MAR002 derivative strain with *LH92*_*RS15870*:: pCR-BluntII-TOPO disruption, Km^r^, Zeo^r^	This work
*LH92_RS15870* complemented	*LH92_RS15870* strain carrying the pBAV1K-T5-gfp plasmid with the corresponding gene cloned, Km^r^, Zeo^r^, Gm^r^	This work
*LH92_RS16065*	MAR002 derivative strain with *LH92*_*RS16065*::pCR-BluntII-TOPO disruption, Km^r^, Zeo^r^	This work
*LH92_RS16065* complemented	*LH92_RS16065* strain carrying the pBAV1K-T5-gfp plasmid with the corresponding gene cloned, Km^r^, Zeo^r^, Gm^r^	This work
*LH92_RS16130*	MAR002 derivative strain with *LH92*_*RS16130*::pCR-BluntII-TOPO disruption, Km^r^, Zeo^r^	This work
*LH92_RS16130* complemented	*LH92_RS16130* strain carrying the pBAV1K-T5-gfp plasmid with the corresponding gene cloned, Km^r^, Zeo^r^, Gm^r^	This work
*LH92_RS16880*	MAR002 derivative strain with *LH92*_*RS16880*::pCR-BluntII-TOPO disruption, Km^r^, Zeo^r^	This work
*LH92_RS17175*	MAR002 derivative strain with *LH92*_*RS17175*::pCR-BluntII-TOPO disruption, Km^r^, Zeo^r^	This work
*LH92_RS17175* complemented	*LH92_RS17175* strain carrying the pBAV1K-T5-gfp plasmid with the corresponding gene cloned, Km^r^, Zeo^r^, Gm^r^	This work
**Plasmids**		
pCR-BluntII-TOPO	Cloning vector, Km^r^, Zeo^r^	Invitrogen
pBAV1K-T5-gfp	Complementation vector, Km^r^	Addgene
pVRL1	Plasmid carrying a gentamicin cassette, Gm^r^	^[38]^
pBAV1Gm-T5-gfp	pBAV1K-T5-gfp derivative vector carrying gentamicin cassette, Gm^r^	This work

Rif^r^, Km^r^, Zeo^r^, Gm^r^ stand for resistance to rifampin, kanamycin, zeocin and gentamicin, respectively.

### Motility assays

The assays were conducted on fresh-agarose plates (0.5% tryptone, 0.25% NaCl, and 0.3% low-agarose; Nzytech) prepared on the day of the experiment and using bacterial cultures at the early stationary phase. A Kolle loop was used to inoculate the plates, followed by gently swirling the medium until the plastic surface of the plate was covered. Bacterial growth occurred at the agarose-plastic interface. The inoculated plates were incubated at either 30°C or 37°C for 16–24 h. Twitching movement was observed at the agarose-plastic interface as a faint bacterial growth halo, and surface-associated motility on the plate surface as a densely branched bacterial halo with irregular edges. All assays were carried out at least three times in independent experiments, each set up in triplicate. Representative images were obtained using a ChemiDoc^TM^ XRS+ system (Bio-Rad).

### Galleria mellonella *killing assays*

The virulence of the *A. baumannii* strains was determined using the *G. mellonella* (wax worm) model, as described previously [33]. Briefly, ten caterpillars were inoculated via the hemocoel with 10 μL of the corresponding *A. baumannii* strain (~ 10^5^ CFU). The inoculants were prepared from exponentially growing cultures (OD_600_ = 0.1) corresponding to ~ 10^8^ CFU/mL and previously diluted in phosphate-buffered saline (PBS). The concentration of each inoculum was confirmed by colony counts on LB plates. As a negative control, the same number of caterpillars was inoculated with 10 μL of PBS. The control and treated caterpillars were incubated at 37°C in the dark and their survival was checked every 12 h for a total of 96 h. All *G. mellonella* killing experiments were performed at least three times.‬‬‬‬‬‬‬‬‬‬‬‬‬‬‬‬‬‬‬‬‬‬‬‬‬‬‬‬‬‬‬‬‬‬‬‬‬‬‬‬‬‬‬‬‬‬‬‬‬‬‬‬‬‬‬‬‬‬

### RNA extraction

A High Pure RNA isolation kit (Roche) was used to isolate RNA from cultures of *A. baumannii* strain MAR002 and the corresponding Rif^r^ mutants, according to the manufacturer’s instructions. Prior to RNA extractions, bacteria growing in LB medium until the mid-exponential growth phase (OD_600_ = 0.4) were pelleted and then treated with lysozyme (50 mg/mL; resuspended in Tris-EDTA (TE) buffer) for 10 min at 37°C. To remove DNA contaminants, all RNA samples were incubated with DNase Turbo (Ambion). DNA removal was confirmed in PCRs using RNA samples.

### RNA-seq

Transcriptomic analysis of *A. baumannii* strain MAR002 WT and the Rif^r^ mutants was performed by Allgenetics (A Coruña, Spain). Briefly, each strain was grown in LB until an OD_600_ of 0.4–0.6, pelleted, and then stored at −80°C until used in RNA-seq. RNA was extracted as described above and its purity was analyzed using a 2100 Bioanalyzer (Agilent). To prepare each library, the ribosomal RNA was removed using the Ribo-Zero Plus rRNA depletion kit (Illumina). The RNA was then processed using the TruSeq Stranded mRNA library prep kit (Illumina), with the fragment size distribution checked using the High Sensitivity DNA kit (Agilent). The libraries were quantified using the Qubit dsDNA HS assay kit (Thermo Fisher Scientific) and pooled in equimolar amounts according to the Qubit results. The resulting pool was sequenced in a HiSeq 4000 PE100 lane (Illumina).

After RNA-seq, the quality of the raw sequencing data was checked, and the trimmed and preprocessed reads were mapped to the *A. baumannii* MAR002 reference genome (NZ_JRHB01000001.1 and NZ_JRHB01000002.1) using STAR 2.6.0a[34]. Read counts were quantified, normalized, and filtered following the trimmed mean of M-values method[35]. Differential gene expression between *A. baumannii* MAR002 WT and the corresponding Rif^r^ mutants was analyzed using the Bioconductor packages NOISeq and NOISeqBIO. The expression of a gene was considered to be induced or repressed when the fold change of the Rif^r^ mutants compared to the parental strain was ≥ 2 or ≤ −2, respectively, and the *P* value of the difference compared to the control was < 0.05. A Venn diagram was constructed using the BxToolBox program available at bioinforx.com.

RNAseq data are available at NCBI (National Center for Biotechnology Information; https://www.ncbi.nlm.nih.gov/) under the accession number PRJNA479736, where Rif7, Rif11 and Rif12 correspond to T^−^S^−^, T^+^S^−^ and T^−^S^+^ strains, respectively.

### Gene expression

To corroborate the transcriptomic data, gene expression was determined by RT-qPCR as previously described[36]. RT-qPCR was conducted using Lightcycler RNA Master SYBR green I (Roche) and a Lightcycler 480 instrument (LC480; Roche) according to the manufacturer’s instructions. The oligonucleotides listed in Table S1 were used to validate the expression of the selected genes identified by RNA-seq. The relative mRNA concentrations of the genes of interest were determined according to a standard curve generated by amplifying an internal fragment of the *gyrB* gene that was not induced under the tested growth conditions. The expression factor was calculated as the ratio of the mRNA concentration of the target gene as expressed by the WT vs. by the studied mutant strain.

### A. baumannii *knockout construction and complementation*

Gene inactivation was carried out by gene disruption, as previously described[37]. Briefly, an internal fragment (from ~ 0.2 to 0.6 kb) of the target gene from *A. baumannii* strain MAR002 WT was PCR-amplified using the corresponding intF and intR primers listed in Table S1. The resulting fragment was cloned into the kanamycin- and zeocin-resistant plasmid pCR-BluntII-TOPO (Invitrogen), which is unable to replicate in *A. baumannii*. The construct was introduced into *E. coli* DH5α by electroporation and selected on kanamycin-containing LB plates. The purified plasmid was then introduced into WT *A. baumannii* by electroporation and the resulting transformants were selected on kanamycin-containing plates. Recombinant clones were confirmed by sequencing (Macrogen) of the PCR products amplified using the appropriated combination of primers (Table S1), one external to the construction (ComFw or ComRv) and the other within the suicide plasmid (M13FpUC or M13RpUC), according to the insert orientation relative to the vector. The stability of the *A. baumannii* knockouts was determined in ten passages without selective pressure, with colony resuspension carried out every 24 h, followed by colony counting on LB plates with and without kanamycin at the last passage.

For mutant complementation, the target ORF was cloned into the pBAV1Gm-T5-gfp plasmid using the appropriate primers (Table S1), which included *Xba*I restriction sequences. The pBAV1Gm-T5-gfp derivative plasmid was generated from the pBAV1K-T5-gfp vector (a gift from Ichiro Matsumura; Addgene plasmid 26,702), in which the kanamycin sequence is substituted by the gentamicin cassette from the pVRL1 plasmid[38]. The recombinant plasmid was introduced by electroporation, firstly into *E. coli* strain DH5α and then, once the correct construct had been verified by PCR and sequencing (Macrogen), into the corresponding *A. baumannii* knockout. The complemented mutants were selected on kanamycin- and gentamicin-containing plates. To assess plasmid stability in the complemented mutants, the cells were plated on solid medium with or without gentamicin.

### Statistical analysis

The data were analyzed in a two-tailed, one-way analysis of variance (ANOVA) followed by the Tukey test for post-hoc multiple-group comparisons. Survival curves obtained in the *G. mellonella* killing assays were plotted using the Kaplan-Meier method and differences in survival were calculated using the log-rank test. In all cases, statistical significance was defined as a *P* value < 0.05.

## Results

### *Twitching and surface-associated motility profiles of Rif^r^ mutants of* A. baumannii *strain MAR002 and the effects on virulence*

As noted above, mutations in the *rpoB* gene of *A. baumannii* ATCC 17,978 change the promoter affinity of the bacterium’s RNA polymerase, which in turn affects several phenotypic properties including motility[23]. To analyze whether mutations in the *rpoB* gene of *A. baumannii* MAR002, a strain able to move through twitching and surface-associated motility, affect both motility systems, saturated cultures of this strain were plated in the presence of rifampin. Several spontaneous *A. baumannii* MAR002 Rif^r^ mutants were isolated and their motility patterns were evaluated.

**Figure 1.**
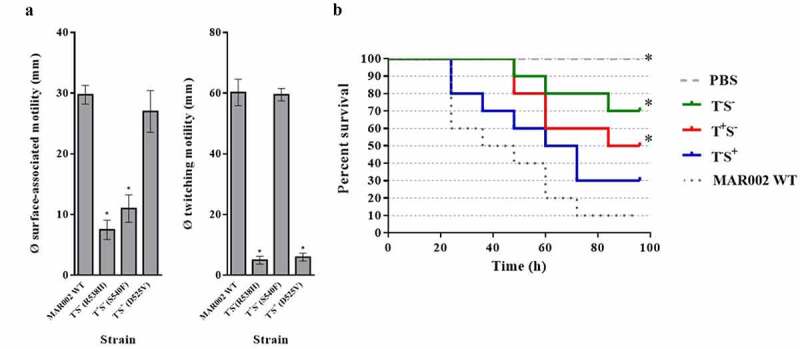
(a) Diameter (in mm) of the halos formed by the indicated strains during both surface-associated motility and twitching. Each experiment was carried out in triplicate in a minimum of three independent motility assays. A significant decrease in motility (P < 0.05) compared to the parental strain is indicated with an asterisk (*). (b) Representative results of a *Galleria mellonella* killing assay using the specified strains. Larvae (n = 10 per group) were inoculated with ~ 10^5^ CFU of the indicated *A. baumannii* strain or PBS as a negative control. A significant decrease in virulence (*P* < 0.05) compared to the parental strain is indicated with an asterisk (*)

Four different movement phenotypes were identified in the mutants: (i) 2.3% impaired in both movement types (T^−^S^−^), (ii) 4.5% impaired in twitching motility (T^−^S^+^), (iii) 25% impaired in surface-associated motility (T^+^S^−^), and (iv) 68.2% without a difference compared to the WT. A representative from each phenotype lacking one or both types of motility was selected (T^−^S^−^: Arg538His; T^−^S^+^: Asp525Val; and T^+^S^−^: Ser540Phe) for further analysis and its *rpoB* gene was sequenced (Figure 1a). To confirm that the motility impairments were due to point mutations located in the *rpoB* gene, further spontaneous Rif^r^ mutants were isolated in which the specific point mutations in the *rpoB* sequence were located in the same positions as obtained previously. These mutants showed that the same mutation in different isolates always implied the same motility phenotype (data not shown). No significant differences in the motility of *A. baumannii* MAR002 at 30°C vs. 37°C were observed (data not shown).

To evaluate the role of each form of motility in the pathogenesis of *A. baumannii* MAR002, the virulence of each of the three types of Rif^r^ mutant with altered motility (T^−^S^−^, T^−^S^+^ and T^+^S^−^) was determined in *G. mellonella* killing assays (Figure 1b). Compared to the WT strain, the virulence of the strains with impaired surface-associated motility (T^−^S^−^ and T^+^S^−^) was significantly (*P* < 0.05) reduced whereas this was not the case for the Rif^r^ mutant with impaired twitching motility alone (T^−^S^+^). After 96 h of incubation, the survival of worms inoculated with mutants lacking surface-associated motility was 70% (T^−^S^−^) and 50% (T^+^S^−^) while that in the worms inoculated with either the T^−^S^+^ mutant or the WT strain was 30% and 10%, respectively (Figure 1b).

### *Identification of* A. baumannii *strain MAR002 Rif^r^ genes involved in motility*

Global gene expression of the Rif^r^ mutants with altered motility (T^−^S^−^, T^−^S^+^, and T^+^S^−^) was characterized by numerous significant changes compared to that of WT *A. baumannii* MAR002. Based on two-fold differences in the up- or down-regulation of gene expression, 771 genes were differentially expressed in the T^−^S^−^ strain, of which 282 were up-regulated and 489 down-regulated (Table S2). Furthermore, 970 genes were differentially expressed in the T^−^S^+^ strain, 489 of which were up-regulated and 481 down-regulated. Finally, 700 genes were differentially expressed in the T^+^S^−^ strain, of which 285 were up-regulated and 415 down-regulated (Table S2). These results were validated in an RT-qPCR analysis comparing selected down-regulated genes from each of the three Rif^r^ mutants with the respective WT genes from the parental strain (Figure S1).

**Figure 2.**
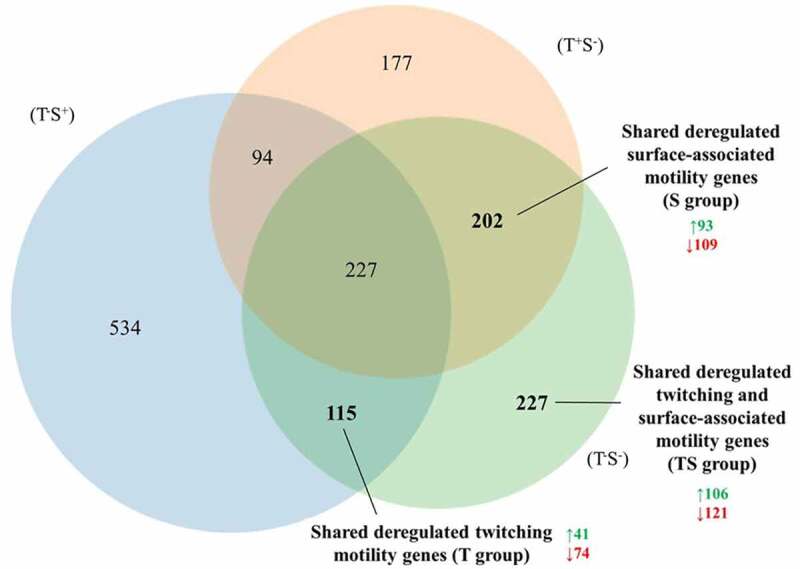
Venn diagram of the distribution of deregulated genes in *A. baumannii* strains T^−^S^−^, T^+^S^−^, and T^−^S^+^. Overlapping areas represent deregulated genes identified in two or all three strains, and the numbers in bold the group of genes hypothetically involved in surface-associated motility, twitching motility, or both in *A. baumannii* strain MAR002. In the indicated groups, the total number of up-regulated (green) or down-regulated (red) genes is also shown

To obtain insights into the involvement of the deregulated genes in twitching and surface-associated motility, they were grouped according to their presence or absence in the Rif^r^ mutants as determined from the RNA-seq analyses (Figure 2 and Table S2). The identification of 115 genes shared by strains with impaired twitching motility (T^−^S^+^ and T^−^S^−^; T group) and 202 genes by strains with impaired surface-associated motility (T^+^S^−^ and T^−^S^−^; S group) implied that these groups of genes, along with those exclusive to strain T^−^S^−^ (227 genes), were involved in twitching motility (T group), in surface-associated motility (S group), or in both (TS group) (Table S2).

Eighteen genes (6 per group) from the three groups (T, S, and TS) were selected to study their possible direct roles in twitching or surface associated motility in the WT strain of *A. baumannii*. The selection was based on the expression levels of these genes, their predicted function with respect to either twitching or surface-associated motility, and their functional group, with the latter including cell wall/membrane/envelope biogenesis, motility, transport, metabolism, and transcription (Table 2).

**Table 2.** Downregulated genes analyzed in this study

	Gene	ID. Protein	Description	Log2 Fold change		
				T^−^S^−^	T^+^S^−^	T^−^S^+^
**TS group**	*LH92_RS01675*	WP_001072475.1	HlyD family secretion protein	−4.50		
	*LH92_RS02585*	WP_000537695.1	Type IV pilus biogenesis/stability protein PilW	−1.03		
	*LH92_RS07135*	WP_000229914.1	Malonate transporter subunit MadM	−2.14		
	*LH92_RS15870*	WP_001046417.1	Prepilin-type N-terminal cleavage/methylation domain-containing protein PilE	−1.68		
	*LH92_RS16130*	WP_021510165.1	Efflux RND transporter periplasmic adaptor	−3.90		
	*LH92_RS16880*	WP_045900525.1	Type VI secretion system protein VgrG	−2.00		
**S group**	*LH92_RS00045*	WP_047479431.1	ABC transporter ATP-binding protein	−2.64	−2.13	
	*LH92_RS05285*	WP_079267361.1	Amino acid permease	−4.66	−2.55	
	*LH92_RS08005*	WP_001162376.1	Phosphoglycerate mutase family protein	−5.93	−3.99	
	*LH92_RS12010*	WP_004840631.1	Multidrug efflux RND transporter outer membrane subunit AdeH	−2.69	−1.10	
	*LH92_RS16065*	WP_000521916.1	Sulfate ABC transporter substrate-binding protein	−3.45	−1.30	
	*LH92_RS17175*	WP_161782838.1	PQQ-dependent sugar dehydrogenase	−4.10	−1.72	
**T group**	*LH92_RS04440*	WP_000837758.1	Energy transducer TonB	−1.81		−6.48
	*LH92_RS05405*	WP_125535337.1	Outer membrane porin family OprD	−1.28		−1.22
	*LH92_RS06300*	WP_045899602.1	Type VI secretion system-associated protein TagF	−1.07		−1.17
	*LH92_RS06715*	WP_000996083.1	IclR family transcriptional regulator	−1.38		−6.70
	*LH92_RS13540*	WP_000669945.1	Hypothetical protein (putative lyase)	−1.30		−7.77
	*LH92_RS13935*	WP_001174793.1	Multidrug efflux RND transporter outer membrane subunit AdeK	−2.01		−1.73

### *Construction and motility characterization of* A. baumannii *knockout mutants of the selected genes*

**Figure 3.**
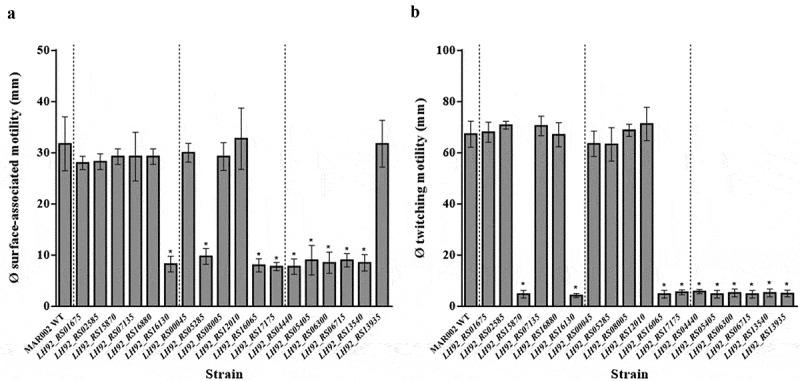
Diameter (in mm) of the halos formed by the indicated strains during surface-associated motility and twitching. Each experiment was carried out in triplicate in a minimum of three independent motility assays. A significant decrease in motility (*P* < 0.05) compared to the parental strain is indicated with an asterisk (*)

To assess whether the selected genes (Table 2) played a role in motility, knockout mutants constructed by gene disruption (Figure S2) were inoculated onto fresh-agarose motility plates. All mutants had a stability of > 90%. The results showed that knockout of the genes encoding the RND component AdeK and the minor prepilin PilE, belonging to the T and TS group, respectively, produced a loss of twitching motility but had no effect on surface-associated motility (Figure 3). Conversely, inactivation of the predicted amino acid permease encoded by the *LH92_RS05285* gene, belonging to the S group, abolished surface-associated motility without altering twitching motility (Figure 3). A complete loss of both twitching and surface-associated motility characterized the knockouts with disruptions in genes belonging to: (i) the T group, including those encoding the energy transducer TonB (which interacts with outer membrane receptor proteins), the porin OprD (an outer membrane protein), the type VI secretion system component TagF, a transcriptional regulator, and a putative lyase; (ii) the S group, including those encoding an ABC transporter component and a PQQ-dependent sugar dehydrogenase; and (iii) the TS group, encoding an RND transporter component (Figure 3).

The remaining knockout mutants (Table 2) showed no alterations in their motility and their behavior was similar to that of the WT strain (Figure 3). Since the growth curves of all mutants grown in LB broth were comparable to that of the parental strain (data not shown), the observed impairments in motility could not be attributed to reductions in the bacterial growth rate.

To demonstrate a direct relation between specific gene disruptions and subsequent movement patterns, the pBAV1Gm-T5-gfp plasmid, carrying the corresponding WT gene, was introduced into all mutants with impaired motility. As expected, complementation consistently restored the parental phenotype (data not shown). Plasmid stability was confirmed in all cases through the methodology specified above.

### *Impaired surface-associated motility but not twitching motility significantly reduces* A. baumannii *virulence*

**Figure 4.**
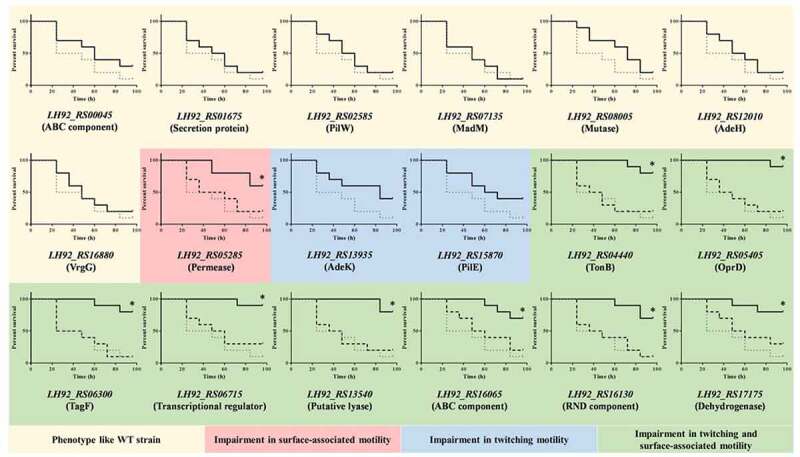
Representative results of a *G. mellonella* killing assay of the specified strains. Larvae (n = 10 per group) were inoculated with ~ 10^5^ CFU of the indicated *A. baumannii* strain. All assays were carried out at least three times. For clarity, the results of a representative assay are presented separately and compared with those obtained with the wild-type (WT) strain (dotted line). In strains marked with an asterisk (*), virulence was significantly reduced (*P* < 0.05) compared with the parental strain (MAR002). In those cases, the survival of worms inoculated with the complemented mutants is also shown (discontinued line). The motility profile of the inoculated strain is indicated by the colored background of each graph

The above-described knockout mutants with altered motility were then studied for their pathogenicity, determined in *G. mellonella* killing assays. Mutants lacking genes involved in surface-associated motility showed a significant (P < 0.05) reduction in their virulence, independent of their twitching behavior (Figure 4). Specifically, > 75% of the worms inoculated with any of the knockout mutants with impairments in both twitching and surface-associated motility (Figure 4) and ~ 60% of those inoculated with the *LH92_RS05285* mutant, with impaired surface-associated motility only, survived until the end of the 96-h experiment (Figure 4). By contrast, virulence was not significantly affected in knockout mutants with impaired twitching but not surface-associated motility (Figure 4). Likewise, knockout mutants with a motility pattern similar to that of the *A. baumannii* MAR002 WT strain did not show a significant reduction in their virulence, as < 25% of the inoculated worms survived. Finally, when the worms were inoculated with the corresponding complemented strains, survival of the larvae was reduced in all cases to the level determined in the WT strain, indicating a restoration of virulence (Figure 4).‬‬‬‬‬‬‬‬‬‬‬‬‬‬‬‬‬

## Discussion

The motility of *A. baumannii* involves two different motility systems: twitching and surface-associated motility. Depending on the strain, one, both, or neither of these movement types is present[18,19]. *A. baumannii* MAR002, a virulent clinical strain, is able to move via both twitching and surface-associated motility [31,39]. In previous work, we showed that spontaneous rifampin-resistant mutants of *A. baumannii* strain ATCC 17,978 carried point mutations in the β-subunit of the RNA polymerase that altered the transcriptional pattern of genes involved in surface-associated motility[23]. In this study, the isolation of spontaneous Rif^r^ mutants in *A. baumannii* strain MAR002 included strains with phenotypic changes in surface-associated and/or twitching motility (Figure 1a).

A connection between motility and virulence has been demonstrated in several pathogenic bacterial species, among them flagellated bacteria [20,21], bacteria displaying type IV pili[40], and bacteria with appendage-independent motility [8,41,42]. In the *A. baumannii* MAR002 Rif^r^ mutants lacking twitching motility, virulence was slightly but not significantly reduced compared to mutants deficient in surface-associated motility (Figure 1b). Transcriptomic studies have shown an increase in the expression of genes involved in the synthesis of type IV pili during the growth of *A. baumannii* in human serum, thus demonstrating the importance of these appendages during bacteremia [2,7,43].

A transcriptional analysis of the Rif^r^ mutants with the three types of altered motility (Table S2) revealed three groups of genes putatively involved in either one or the other or both motility systems (Figure 2). In *P. aeruginosa*, a phylogenetic neighbor of *A. baumannii*, transcriptomic studies of motile versus non-motile cells revealed an overexpression of genes involved in metabolism and secretion during the development of motility by this species [44,45]. Given the pleiotropic effects produced by *rpoB* gene mutations and the complex regulation of motility, the wide range of deregulated genes identified in the transcriptomic analysis was not surprising. The identified genes included those involved in the above-mentioned processes [23,29,46] as well as genes that co-regulate motility and other biological processes under the control of key bacterial regulators, such as cyclic diguanylate [13,47], chemotaxis [15,16,48], and two-component systems [17,49] (Tables S2 and S3).

The roles in twitching and/or surface-associated-motility of the down-regulated genes selected from the transcriptional analysis of the *rpoB* mutants were directly investigated through the construction of knockouts. Based on the transcriptional data, 18 down-regulated genes belonging to the three gene groups were inactivated (Table 2). In 11 of the derivative knockout mutants, twitching and/or surface-associated motility were reduced (Figure 3). However, although the deregulated genes could be classified according to their putative involvement in twitching motility (T group), surface-associated motility (S group), or both (TS group), not all knockouts exhibited the expected phenotype. These results can be explained by the wide range of deregulated genes that lead to a disturbed motility [25,46], as shown by the transcriptomic data (Table S2), whereas in the knockout mutants only one of these genes was inactivated.

In the virulence assay, the pathogenicity of the strain-MAR002-derived knockout mutants was similar to that of the Rif^r^ mutants and could be linked to the respective motility pattern. Specifically, the pathogenicity of the mutants with a phenotype similar to that of the T^−^S^−^ or T^+^S^−^ strains but not the T^−^S^+^ strain was significantly attenuated (Figures 3 and 4), thus demonstrating the greater participation of surface-associated motility over twitching in the virulence of *A. baumannii*.

In the *A. baumannii* MAR002 Rif^r^ mutants, all of the down-regulated genes identified in this work encode putative proteins related to cell envelope biogenesis, transporters, and metabolic enzymes, i.e., functions that play important roles in motility and/or virulence. From these down-regulated genes, 18 *A. baumannii* knockout mutants were constructed and their motility was then analyzed. In two mutants, in which either the gene encoding the prepilin PilE or the RND transporter component AdeK was inactivated, twitching was impaired whereas in another mutant, encoding an amino acid permease, surface-associated motility was impaired. Furthermore, the inactivation of eight genes, including those encoding RND and ABC components, the energy transducer TonB, the porin OprD, the T6SS component TagF, an IclR transcriptional regulator, a PQQ-dependent sugar dehydrogenase, and a putative pectate lyase, revealed their involvement in both twitching and surface-associated motility.

PilE and AdeK were the only proteins with a demonstrated role in twitching but not in the surface-associated motility of *A. baumannii* strain MAR002. PilE is a prepilin subunit involved in the biogenesis of type IV pili. These long protein polymers participate in twitching motility, attachment, biofilm formation, and DNA uptake [50–52]. In *P. aeruginosa*, PilE and other minor pseudopilins comprise the minimal set of components required for pilus assembly[52], which would also explain why a prepilin subunit is required for the twitching motility of *A. baumannii*. However, the finding of an involvement of AdeK, the outer membrane component of an RND transporter, in twitching was unexpected. The *adeI, adeJ*, and *adeK* genes encode, respectively, the membrane fusion, RND, and outer membrane components of the efflux pump AdeIJK, which participates in the extrusion of several antimicrobials in *A. baumannii*[53]. The absence of AdeK was presumably unrelated to the altered expression of pili whereas the absence of the cytoplasmic transporter AdeJ was previously implicated in the impaired surface-associated motility of *A. baumannii* ATCC 17,978 and *A. nosocomialis*, probably through a disruption in the secretion of the surfactants required for this type of motility [11,54]. Interestingly, it has been reported that the absence of efflux pumps such as MuxABC-OpmB affects twitching in *P. aeruginosa* [55,56]. Thus, the extrusion of some molecules may be required not only for surface-associated motility but also for twitching. For instance, *Myxococcus xanthus* requires extracellular polysaccharides for attachment and for the retraction of type IV pili[57].

The amino acid permease encoded by the *LH92_RS05285* gene was the only component detected in this work that was implicated solely in the surface-associated motility of *A. baumannii*. Other genes, encoding transporter-related components, could be attributed to both twitching and surface-associated motility: *LH92_RS16065, LH92_RS16130, LH92_RS05405*, and *LH92*_*RS04440*, encoding an ABC transporter component, an RND component, the outer membrane protein OprD, and TonB, an energy transducer of transporters, respectively. Protein transporters may be important for motility, through their extrusion of molecules such as surfactants; and pathogenesis, by the secretion of virulence factors. In fact, efflux pumps from at least five of the six different superfamilies described in *A. baumannii* were implicated in the bacterium’s surface-associated motility, presumably related to their role in the secretion of extrapolymeric substances [8,9,11,54]. Similarly, in a recent work by Blaschke *et al*., outer membrane proteins were shown to be involved in the surface-associated motility of *A. baumannii* ATCC 17978^10^. In *P. aeruginosa*, a transposon insertion in the *tonB* gene results in a phenotype that includes defective twitching motility and the reduced assembly of extracellular pili, indicating the involvement of TonB in the transport and secretion of pili or in a component required for their formation[58].

Other proteins shown in this study to participate in both twitching and surface-associated motility were a PQQ-dependent sugar dehydrogenase and a putative pectate lyase, encoded by the genes *LH92_RS17175* and *LH92*_*RS13540*, respectively. Metabolic enzymes promote different types of motility in *A. baumannii*[23] and in other bacterial species, such as *Pseudomonas* spp[59]., *E. coli*[60], and *Xanthomonas oryzae*[61]. PQQ-dependent dehydrogenases play a role in the oxidation of monosaccharides. Although their physiological function is uncertain, the results of several studies suggest that they serve as energy-conserving systems [62–64] in high-energy processes such as motility. By catalyzing the depolymerization of bacterial capsules, the putative pectate lyase could facilitate bacterial displacement. The pectate lyases encoded by bacteriophage are responsible for the depolymerization of polysaccharides to allow their specific binding to *Acinetobacter* bacterial capsules[65].

Two other detected genes were shown to be involved in both twitching and surface-associated motility: *LH92_RS06715* and *LH92_RS06300*. In *A. baumannii, LH92_RS06715* encodes an IclR family transcriptional regulator involved in the degradation of p-hydroxybenzoate in *Acinetobacter* spp[66].. In *Acinetobacter* spp., 2,3-dihydroxybenzoate, an iron binding compound, is also a precursor for more complex siderophores such as acinetobactin[67]. Iron limitation in *A. baumannii* grown under iron-limited conditions results in impaired motility[67], which suggests that the lack of siderophores impacts the motility of this bacterium. Finally, inactivation of *LH92*_*RS06300*, encoding the type VI secretion system-associated protein TagF, also impairs the motility of *A. baumannii* MAR002. A gene cluster predicted to encode a type VI secretion system is upregulated in motile populations of *A. baumannii*[22]. Taken together, these findings suggest that type VI secretion systems significantly contribute to the virulence and motility of *A. baumannii*, as also recently demonstrated in the gram-negative bacteria *Xanthomonas phaseoli*[68].

In summary, this work is the first to identify genes involved in either twitching, surface-associated motility, or both in an *A. baumannii* strain capable of both movement types. The identified genes are mainly involved in cell envelope biogenesis, transport, and metabolism, but they are also clearly associated with motility and virulence. Specifically, genes encoding PilE (LH92_RS15870) and the RND efflux pump component AdeK (LH92_RS13935) were shown to be associated with twitching, and a gene encoding an amino acid permease (LH92_RS05285) was linked to surface-associated motility.

In addition, genes encoding a putative pectate lyase (LH92_RS13540), a PQQ-dependent sugar dehydrogenase (LH92_RS17175), an IclR transcriptional regulator (LH92_RS06715), an efflux RND component (LH92_RS16130), the porin OprD (LH92_RS05405), an energy transducer TonB (LH92_RS04440), an ABC component (LH92_RS16065), and the type VI secretion system component TagF (LH92_RS06300) were found to be associated with both movement types. The transcriptomic profiles described herein constitute a starting point for further investigations of the molecular basis of the pathogenic processes of this multidrug-resistant bacterium and thus for the development of new therapeutic agents aimed at novel targets.

## Supplementary Material

Supplemental Material
