# Supplementary material for: Importance of twitching and surface-associated motility in the virulence of Acinetobacter baumannii
Source: Virulence. 2021 Sep 13;12(1):2201–13. doi: 10.1080/21505594.2021.1950268 (PMC8451467; doi:10.1080/21505594.2021.1950268)
Supplement: Supplemental Material [file KVIR_A_1950268_SM2955.zip › supplementary/SUPPLEMENTARY FIGURE LEGENDS.docx]

**SUPPLEMENTARY FIGURE LEGENDS**

**Figure S1.** Verification of the expression of the indicated genes by qRT-PCR in *A. baumannii* T^−^S^−^, T^+^S^−^, and T^−^S^+^ strains. The expression factor is the ratio of the mRNA concentration of each gene from the indicated strain compared to that of the WT strain of *A. baumannii* MAR002. Error bars represent the standard deviations of the mean of at least three independent experiments.

**Figure S2.** PCR verification of the constructed mutant strains. (A) The oligonucleotides used to verify each of the indicated *A. baumannii* knockouts. The sizes of the PCR products are shown in base pairs (bp). (B) PCR verifications of the indicated *A. baumannii* strains. The NZYDNA ladder VII (Nzytech) was used as a DNA size marker (M).
