# Supplementary material for: Importance of twitching and surface-associated motility in the virulence of Acinetobacter baumannii
Source: Virulence. 2021 Sep 13;12(1):2201–13. doi: 10.1080/21505594.2021.1950268 (PMC8451467; doi:10.1080/21505594.2021.1950268)
Supplement: Supplemental Material [file KVIR_A_1950268_SM2955.zip › supplementary/Supplementary Table 1.docx]

**Supplementary Table 1.** Oligonucleotides used in this work.

| **Name** | **Sequence (5’ to 3’)** | **Application** |
| --- | --- | --- |
| rpoB-1441F | GAGCGTGCTGTTAAAGAGCG | rpoB sequencing |
| rpoB-2095R | CTGCCTGACGTTGCATGT | rpoB sequencing |
| 1675RTFw | GTCTAGCCCAAGCCAAAGCAAACT | RT-qPCR assay |
| 1675RTRv | TTACCCGGACGCAAATCAAAG | RT-qPCR assay |
| 5285RTFw | ATCGGGCTGGAATTATTGGGTAGT | RT-qPCR assay |
| 5285RTRv | AAAAAGGCGAGCATATAAAACAGG | RT-qPCR assay |
| 6300RTFw | AGCCCTGCGCGTGGTGAT | RT-qPCR assay |
| 6300RTRv | GGAAAAGCTCGGCCTGAACTGT | RT-qPCR assay |
| 6715RTFw | GCATTAGCACGTGGTTTAGAGC | RT-qPCR assay |
| 6715RTRv | AGAGCGAATCGATACGTTAGACAG | RT-qPCR assay |
| 7135RTFw | GCGGTGCAATGCTTCGTGACT | RT-qPCR assay |
| 7135RTRv | ACCCGTAACAGGCCCAACAATG | RT-qPCR assay |
| 8005RTFw | AGCCCTGAGCCGAATACACTAC | RT-qPCR assay |
| 8005RTRv | ACCATACAAACAGCCCTTCCATC | RT-qPCR assay |
| 13935RTFw | AGTGCAATTCGTCAGGTTTCTCAA | RT-qPCR assay |
| 13935RTRv | GCTACATCATTACGCGCAGTTTCT | RT-qPCR assay |
| 15870RTFw | TGCTTCAATTGCTATCCCTTCAT | RT-qPCR assay |
| 15870RTRv | AAATTTCCTGTCGTCGTATCATCA | RT-qPCR assay |
| 17175RTFw | ACCCTGCCCAAAATATGAAAAGTA | RT-qPCR assay |
| 17175RTRv | CTGGGCGCGTGTGATGAT | RT-qPCR assay |
| GyrBRTFw | TACAGACGACGGTACCGG | RT-qPCR assay |
| GyrBRTRv | CTGACCGATTCATCTTCG | RT-qPCR assay |
| 0045IntFw | GCGTCTATGCAATTACAC | Mutant construction |
| 0045IntRv | GAATATCCCAATATTCTTC | Mutant construction |
| 0045ComFw | CAGTtctagaATGATTCAATTTGACCAAG | Mutant verification |
| 0045ComRv | CAGTtctagaTTAGTTTTCAAATGAGGC | Mutant verification |
| 1675IntFw | GGAACTACTATAATGCAGCACCG | Mutant construction |
| 1675IntRv | CGGCTAACTTAATGTTAGTAC | Mutant construction |
| 1675ComFw | CAGTtctagaATGAATCAATTGGAGTTG | Mutant verification |
| 1675ComRv | CAGTtctagaTTATTTTTCAATAATATGTAC | Mutant verification |
| 2585IntFw | GAAAAAGCAGTAAAAGTGCG | Mutant construction |
| 2585IntRv | CCAGCAATACGAAATTGCTC | Mutant construction |
| 2585ComFw | CAGTtctagaATGGGGGTTGCTGTTGCAC | Mutant verification |
| 2585ComRv | CAGTtctagaTTATTTCCATACGGCCTCAG | Mutant verification |
| 4440IntFw | GATTGTTATGTTGCCTTTAC | Mutant construction |
| 4440IntRv | CTATTGGCGTTTTGTGTCAACTG | Mutant construction |
| 4440ComFw | CAGTtctagaATGAAATCAAAATCATTATTC | Mutant complementation and verification |
| 4440ComRv | CAGTtctagaTTAACGGCCCCGATTGAGTTTG | Mutant complementation and verification |
| 5285IntFw | CACATCCAGTTAATCGCTATG | Mutant construction |
| 5285IntRv | GTACACTACCCAATAATTCCAG | Mutant construction |
| 5285ComFw | CAGTtctagaTTGAGTAACTCATCTTCCC | Mutant complementation and verification |
| 5285ComRv | CAGTtctagaTTAGAGTGATTTTTTCCAACTC | Mutant complementation and verification |
| 5405IntFw | CTCTGGTTCAGTGTGGGCTGGC | Mutant construction |
| 5405IntRv | CTTCAATGCAGGATTAAACTG | Mutant construction |
| 5405ComFw | CAGTtctagaATGTCGAAATTATGGATGTAC | Mutant complementation and verification |
| 5405ComRv | CAGTtctagaTTATTTAAATTTCCATGTGTAG | Mutant complementation and verification |
| 6300IntFw | GATTTCTTAAAGACAAAAGGTC | Mutant construction |
| 6300IntRv | CCAATCATGCTTTGAGCTAACTC | Mutant construction |
| 6300ComFw | CAGTtctagaATGCTTACATTAAAAACAACTCC | Mutant complementation and verification |
| 6300ComRv | CAGTtctagaTTATTCACCCAAAAAAGTCTG | Mutant complementation and verification |
| 6715IntFw | CAGAAAAATTATTAGCGCC | Mutant construction |
| 6715IntRv | GGCATTGCAGCTAAACATGCAC | Mutant construction |
| 6715ComFw | CAGTgaattcATGAATGATGCAGTTGATGC | Mutant complementation and verification |
| 6715ComRv | CAGTgaattcTTAGCTAATCTCGGTACGAATA | Mutant complementation and verification |
| 7135IntFw | CCAATGGACGTGTGCATGC | Mutant construction |
| 7135IntRv | CCAGCTTTTTTGGCCTCAG | Mutant construction |
| 7135ComFw | CAGTtctagaATGGTTGAATTATTATTAAAAG | Mutant verification |
| 7135ComRv | CAGTtctagaTTAACCCACCAAAGCTTTAAC | Mutant verification |
| 8005IntFw | CTGATGCTTCGACCCCTG | Mutant construction |
| 8005IntRv | GCTACCAGGTTCAACCAAC | Mutant construction |
| 8005ComFw | CAGTtctagaATGGCACTCGATTTATTGCC | Mutant verification |
| 8005ComRv | CAGTtctagaTTACTGTACTTTATGGAAC | Mutant verification |
| 12010IntFw | GCTTCTTGCAGGCTGCTC | Mutant construction |
| 12010IntRv | GCATTGTCATCTAAACCGAG | Mutant construction |
| 12010ComFw | CAGTtctagaGTGATTACATCAAAACAAAAC | Mutant verification |
| 12010ComRv | CAGTtctagaTTAACTACTCCAACCGCCCCCT | Mutant verification |
| 13540IntFw | CTGCTCATCTGCATTTGCTC | Mutant construction |
| 13540IntRv | GATGCATTCAAAGTATCAACAG | Mutant construction |
| 13540ComFw | CAGTtctagaATGAAATTTAAATACTTATC | Mutant complementation and verification |
| 13540ComRv | CAGTtctagaTTATTGGACCCGACTTATTTTGATC | Mutant complementation and verification |
| 13935IntFw | CTGCACTTGCGCTTGCTTTGGC | Mutant construction |
| 13935IntRv | CGAATTGCACTACCACTTGC | Mutant construction |
| 13935ComFw | CAGTtctagaATGCAAAAAGTATGGTCTATTTC | Mutant complementation and verification |
| 13935ComRv | CAGTtctagaTTATTGCTTTTTAAGTTCAGC | Mutant complementation and verification |
| 15870IntFw | GCTATCCCTTCATATCAAAG | Mutant construction |
| 15870IntRv | CGTCAATAAATTTCCTGTCG | Mutant construction |
| 15870ComFw | CAGTtctagaATGAATAAGTACTCTATTC | Mutant complementation and verification |
| 15870ComRv | CAGTtctagaTCATTGCCAATTCTCACTATTTG | Mutant complementation and verification |
| 16065IntFw | CAGTAAGTGCTGTGCAGGC | Mutant construction |
| 16065IntRv | GCCTGCTTTTACAATTTCATC | Mutant construction |
| 16065ComFw | CAGTtctagaATGAAAAGAATAGCAATAATTG | Mutant complementation and verification |
| 16065ComRv | CAGTtctagaCTACTGCTTTCCATCATAAATC | Mutant complementation and verification |
| 16130IntFw | GAACAAGCTGAACTAGGTG | Mutant construction |
| 16130IntRv | GTTCACGCTCATAGTCCTGACG | Mutant construction |
| 16130ComFw | CAGTtctagaATGTCGGCAAATTTAAAGAA | Mutant complementation and verification |
| 16130ComRv | CAGTtctagaTTAATGTCCATGCTCAGCCTC | Mutant complementation and verification |
| 16880IntFw | GACTGAGGCCAGTTATGGAC | Mutant construction |
| 16880IntRv | CGCCACAAACGTGTGAGAAAG | Mutant construction |
| 16880ComFw | CAGTtctagaATGCTTTTTAATATATTTAG | Mutant verification |
| 16880ComRv | CAGTtctagaTTATACTCCAAATTTATTAAAATC | Mutant verification |
| 17175IntFw | GGCCTGTGGCTCTAATAATAC | Mutant construction |
| 17175IntRv | GTGATTTTTGGCAAAGTC | Mutant construction |
| 17175ComFw | CAGTtctagaATGAGACGATTAGCAGCCCCCT | Mutant complementation and verification |
| 17175ComRv | CAGTtctagaTTAAGAGGGTTTCTTACTC | Mutant complementation and verification |
| M13FpUC | GTTTTCCCAGTCACGAC | Sequencing primer for pCR-BluntII-TOPO |
| M13RpUC | CAGGAAACAGCTATGAC | Sequencing primer for pCR-BluntII-TOPO |
| Gm^r^Fw | GAGTCCAAGCGAGCTCGGTATTAGGTGGCGGTACTTGG | Amplification of the gentamicin cassette from pVRL1 |
| Gm^r^Rv | AAAGAGGAAGGAAATAATAAATGTTACGCAGCAGCAAC | Amplification of the gentamicin cassette from pVRL1 |
| pBAV1KInvFw | TTATTATTTCCTTCCTCTTTTCTACAGTATTTAAAG | Amplification of the pBAV1K-T5-gfp vector |
| pBAV1KInvRv | TACCGAGCTCGCTTGGAC | Amplification of the pBAV1K-T5-gfp vector |
| pBAV1KExtFw | ATCCAGATGGAGTTCTGAGGT | pBAV1Gm-T5-gfp vector verification |
| pBAV1KExtRv | CGTCTTGTTATAATTAGCTT | pBAV1Gm-T5-gfp vector verification |
| pBAV1Fw | GACGAACTCCAATTCACTGTTCCTTGC | Sequencing primer for pBAV1Gm-T5-gfp |
| pBAV1Rv | ATTGGGACAACTCCAGTGAA | Sequencing primer for pBAV1Gm-T5-gfp |

Restriction endonuclease sites are indicated in lower case.
