# Supplementary figures and images for: Importance of twitching and surface-associated motility in the virulence of Acinetobacter baumannii
Source: Virulence. 2021 Sep 13;12(1):2201–13. doi: 10.1080/21505594.2021.1950268 (PMC8451467; doi:10.1080/21505594.2021.1950268)

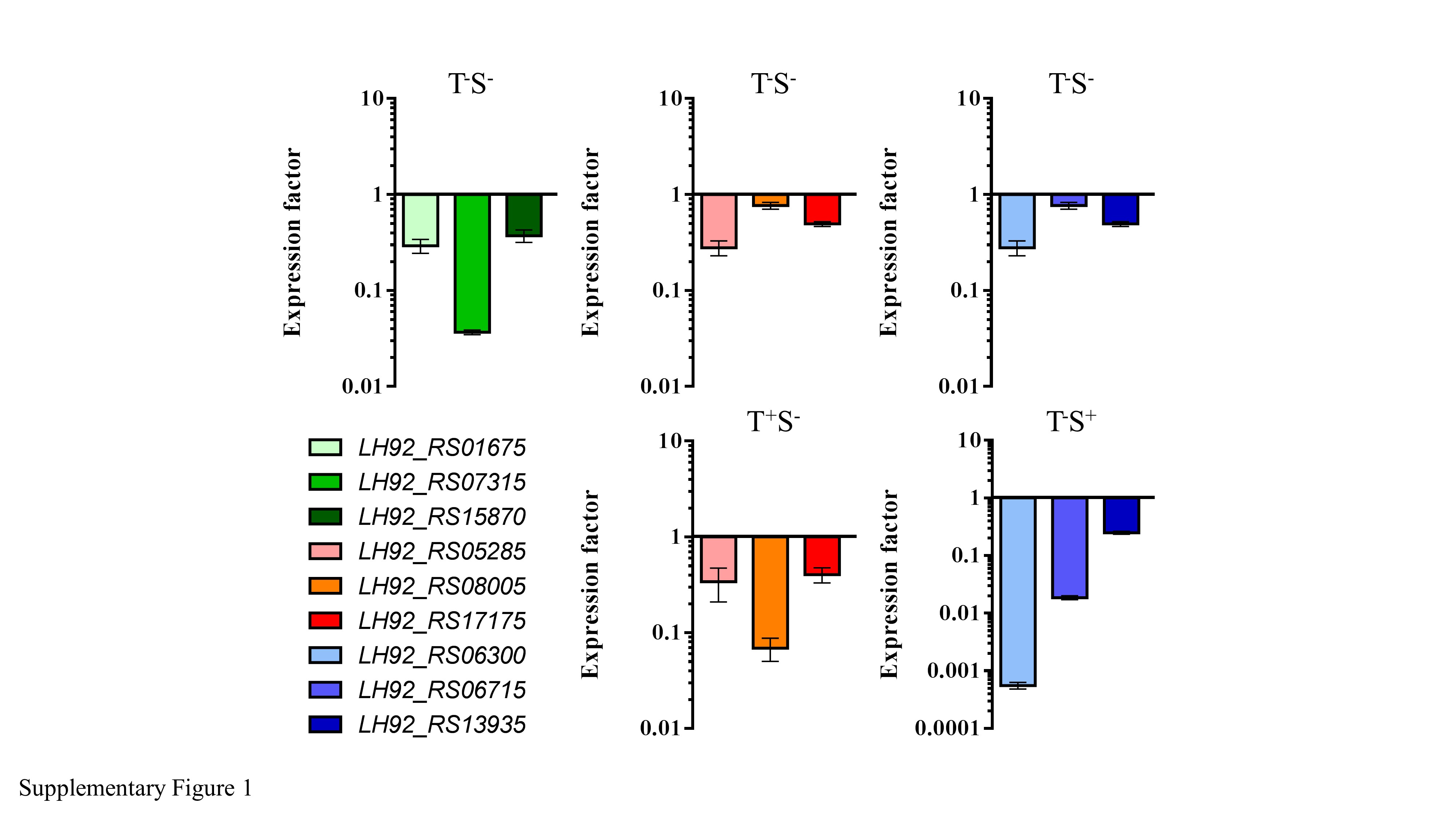

Supplement: Supplemental Material [file KVIR_A_1950268_SM2955.zip › supplementary/Supplementary Figure 1.JPG]

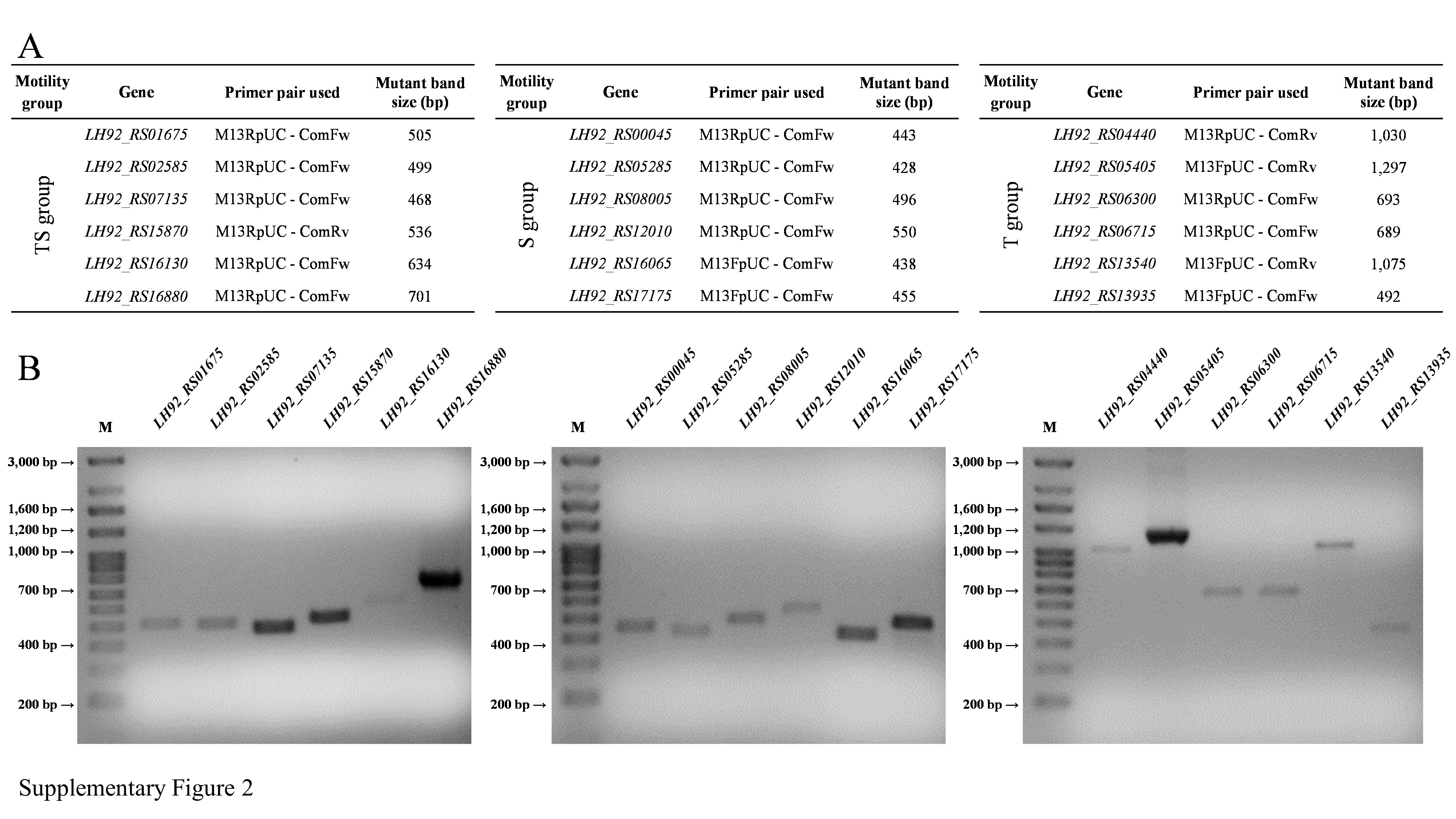

Supplement: Supplemental Material [file KVIR_A_1950268_SM2955.zip › supplementary/Supplementary Figure 2.JPG]
